# Amoebic liver abscess co-infected with bacterial liver abscess: A rare case in an immunocompromised patient

**DOI:** 10.1016/j.idcr.2025.e02279

**Published:** 2025-06-06

**Authors:** Zhuo-Lin Zou, Zhong-Hai Shen

**Affiliations:** a Department of Infectious Diseases, The First Hospital of Jiaxing, Affiliated Hospital of Jiaxing University, Jiaxing 314000, China; b Institute of Hepatology, Affiliated Hospital of Jiaxing University, Jiaxing 314000, China; c Department of Orthopedic Surgery, The Second Affiliated Hospital of Jiaxing University, Jiaxing, China

**Keywords:** Amoebic liver abscess, Bacterial liver abscess, HIV, mNGS

## Abstract

We present a complex case of a 43-year-old HIV-positive Chinese male with co-infection of amoebic liver abscess (ALA) and bacterial liver abscess caused by *Salmonella enterica* subsp. *enterica* serotype Typhi (abbreviated as *Salmonella* Typhi). The patient presented with fever and abdominal pain. Initial bacterial cultures identified *Salmonella* Typhi, but targeted antibiotic therapy failed to resolve his symptoms, prompting to further investigation. Metagenomic next-generation sequencing (mNGS) of pleural and liver abscess drainage fluids revealed sequences of *Entamoeba histolytica*, confirming a dual infection. The patient was treated with combination therapy, resulting in clinical improvement. This case highlights diagnostic challenges in immunocompromised patients and underscores the critical role of mNGS in identifying co-infections and guiding treatment. Early recognition and timely intervention are essential for achieving optimal outcomes in such complex cases.

## Introduction

Amebiasis, caused by *Entamoeba histolytica*, is transmitted via ingestion of food or water contaminated with the cyst form of the parasite, leading to amebic colitis and amebic liver abscess formation [1]. It is prevalent in developing countries, including Central and South America, tropical Asia, and Africa [2]. The severity of the disease can be influenced by various host factors, including immune status, pregnancy, malnutrition, genetic characteristics, and gender [3]. Most infections (90 %) are asymptomatic, and the remaining 10 % produce a spectrum of clinical syndromes ranging from dysentery to abscesses in the liver or other organs, with less than 1 % of patients developing an invasive infection [4], [5].

Extraintestinal infections caused by *Entamoeba histolytica* most commonly involve the liver [6]. ALA are more frequently found in the right lobe of the liver and usually present as solitary lesions, pleuropulmonary involvement is the most common complication [7]. Diagnosis relies on stool examination, serological testing, and noninvasive liver imaging. However, underdiagnosis is common in non-endemic areas due to limited diagnostic tools and low disease awareness [8].

It is important to note that liver abscesses can also be caused by bacterial and fungal pathogens, each with their own set of risk factors. For instance, bacterial liver abscesses are often associated with biliary tract issues, uncontrolled diabetes, or cirrhosis, and can result from the spread of intra-abdominal infections [9]. Fungal liver abscesses, on the other hand, primarily affect immunocompromised individuals [10]. Additionally, co-infections involving bacteria and *Entamoeba* species have been reported in the literature. For example, co-infection with bacteria has been observed in amoebic liver abscesses, and co-infection between *Entamoeba* and *Salmonella* typhi has been reported in intestinal infections [11], [12]. These findings highlight the complexity of diagnosing and treating liver abscesses, as they may involve multiple pathogens. We present a case of an ALA co-infected with a bacterial liver abscess in an HIV-positive patient, emphasizing diagnostic and management challenges.

## Case presentation

In March 2023, a 43-year-old Chinese male presented to the fever clinic with a one-week history of fever (peak temperature 39.8 °C) and right upper quadrant abdominal pain. He had no significant medical history and denied corticosteroid or immunosuppressive drug use. On admission, the patient's vital signs were as follows: pulse rate 119 beats/min, respiratory rate 18 breaths/min, blood pressure 112/69 mmHg, and body temperature 38.5℃. Laboratory tests showed normal white blood cell count (6.72 × 10⁹/L; reference range, 3.5–9.5 × 10⁹/L) but elevated neutrophil ratio (77.5 %; reference range, 40–75 %) and C-reactive protein (78.9 mg/L; reference range, 0–8 mg/L), liver function tests were within normal limits: Alanine aminotransferase (ALT) (32IU/L, reference range, 9–50 IU/L), Aspartate aminotransferase (AST) (40 IU/L, reference range, 15–40 IU/L).

**Fig. 1:**
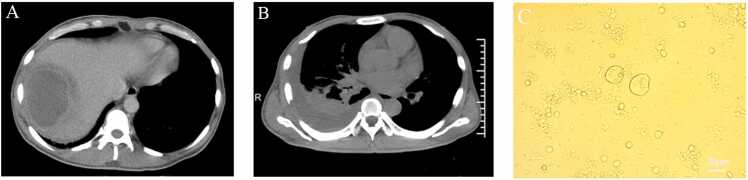
Amoebic liver abscess co-infected with bacterial liver abscess in an HIV patient. (A) Enhanced abdominal CT scan showing a hypodense lesion in the right lobe of the liver with peripheral ring enhancement. (B) Chest CT scan revealing a large right-sided pleural effusion and incomplete expansion of the right lower lobe. (C) The sample was prepared using the wet mount method without any staining. Microscopic examination of the drainage fluid showing unicellular organisms with pseudopods.

Following evaluation at the fever clinic, he was admitted to the infectious disease department. Laboratory results revealed a positive HIV antibody test, the Jiaxing Municipal Center for Disease Control and Prevention (Jiaxing CDC: responsible for disease surveillance and public health management at the municipal level) confirmed the positive result, further laboratory tests revealed a CD4 count (163.4 cells/μL, reference range, 324.60–1274.70 cells/μL) and viral load (153,270 copies/mL, reference range, < 20 copies/mL). Enhanced abdominal CT scan revealed low-density areas in the right lobe of the liver, with a CT value of approximately 30 Hounsfield Unit (HU) and peripheral ring enhancement observed after the administration of contrast material, suggestive of an abscess (Fig. 1, panel A).

The patient had a persistent high fever, prompting ultrasound-guided percutaneous drainage of the hepatic abscess (PLAD), which yielded a large amount of pus. Bacterial culture of the drained fluid (aerobic and anaerobic) and pre-antibiotic blood cultures (four bottles: 2 aerobic, 2 anaerobic from bilateral arms; three positive: both aerobic, left anaerobic) identified *Salmonella enterica* subsp. *enterica* serotype Typhi (abbreviated as *Salmonella* Typhi: identified by biochemical tests (API 20E) and confirmed via MALDI-TOF MS). Using broth microdilution (MIC) and Kirby-Bauer (zone diameter) methods per CLSI 2023 guidelines, the isolate demonstrated susceptibility to: ceftazidime (MIC ≤ 1 μg/mL), cefepime (MIC ≤ 1 μg/mL), trimethoprim-sulfamethoxazole (MIC ≤ 1/19 μg/mL), piperacillin-tazobactam (MIC 8 μg/mL), and aztreonam (MIC ≤ 1 μg/mL). Antimicrobial therapy with ceftriaxone 2000 mg every 24 h was initiated. However, despite continuous drainage of the liver abscess and targeted anti-*Salmonella* therapy, the patient continued to have a fever and gradually developed dyspnea. Chest and abdominal CT scans (on the 14th day of hospitalization) showed enlargement of the liver abscess and rupture into the pleural space (Fig. 1, panel B).

Further inquiry into his medical history revealed that the patient had a history of diarrhea and abdominal pain two months before presentation, which resolved spontaneously without treatment, and He originated from an amebiasis-endemic region in China and denied special social history.

Unfortunately, no hospitals or laboratories in southern China currently offer amoebic serological testing. Given the patient's pleuropulmonary involvement and CT findings suggestive of abscess rupture into the pleural space, an ultrasound-guided thoracentesis was performed (on the 14th day of hospitalization), which yielded 1500 mL of a "fish sauce-like" fluid, on the 16th day of hospitalization, mNGS of both pleural and liver abscess drainage fluids detected sequences of *Entamoeba histolytica.* Additionally, mNGS of the liver abscess drainage fluid revealed sequences of *Salmonella enterica* subsp. *enterica* serotype Typhi (abbreviated as *Salmonella* Typhi) and *Salmonella enterica* subsp. *enterica* serotype Typhimurium (abbreviated as *Salmonella* Typhimurium), then, rapid wet mount microscopic examination of additional liver drainage from drainage tubes fluid revealed unicellular organisms with pseudopods, suggestive of amoebic trophozoites (Fig. 1, panel C)

Combining findings from microscopic examination of the drainage fluid, microbiological culture, radiological imaging, and mNGS results, the diagnosis was confirmed as an amoebic liver abscess complicated by concurrent *Salmonella* Typhi and *Salmonella* Typhimurium bacterial liver abscesses. Although gastrointestinal endoscopy was recommended by the medical team due to the patient's prior intestinal symptoms, the patient declined further evaluation. However, the possibility of intestinal amoebic infection could not be ruled out. Consequently, the patient was treated with a 10-day regimen of metronidazole (750 mg every 8 h), 26-day ceftriaxone (2000 mg once daily), and paromomycin (500 mg three times daily, initiated 16 days after admission for a 10-day course.) to eradicate cysts in the intestinal lumen.

After treatment, the patient's symptoms resolved, and he was discharged in stable condition. Based on the antibiotic susceptibility testing of the *Salmonella* Typhi isolate from the aspirate culture, the patient continued oral trimethoprim-sulfamethoxazole for four weeks after discharge until inflammatory markers normalized completely and follow-up imaging confirmed complete resolution of the abscess.

## Discussion

Amebiasis, caused by *Entamoeba histolytica*, is the third leading cause of death from parasitic infections worldwide[13]. Most infected individuals are asymptomatic carriers. In the general population, the incidence of liver abscess varies by region, but in HIV-positive individuals, there is a heightened susceptibility to atypical pathogens. HIV-positive patients are more likely to experience invasive lesions caused by E. histolytica [14]. Pleuropulmonary complications, which occur in 15–20 % of ALA cases, are a significant extrahepatic concern. These complications often arise from the spread of the abscess to the right hepatic lobe near the diaphragm, leading to pleural effusion, empyema, or bronchopleural fistula, and sometimes lung abscesses. The symptoms can mimic those of bacterial pneumonia or tuberculosis, making diagnosis challenging in non-endemic areas [15].

In our case, the patient originated from an amebiasis-endemic region in China and had a suspected history of intestinal amebiasis prior to presentation. However, gastrointestinal endoscopy was declined, making clinical confirmation impossible. We speculate that the patient ingested infectious *E. histolytica* cysts while in the endemic area, leading to subsequent symptoms that resolved spontaneously. As an immunocompromised individual with acquired immunodeficiency, the patient experienced a decline in CD4+ T cells, contributing to disease progression. However, concurrent *Salmonella* Typhi and Typhimurium complicate the etiology, as both pathogens independently or synergistically provoke gastrointestinal inflammation [11]. While *Salmonella* Typhimurium typically causes self-limited enteritis, co-infections may amplify colonic damage [16]. The lack of colonoscopy obscured definitive mucosal assessment, leaving symptom attribution unresolved, paromomycin was empirically administered to target residual *E. histolytica* cysts, aligning with the patient’s prolonged symptoms and endemic exposure. This reflects pragmatic, syndromic management in resource-limited settings, where overlapping infections demand context-guided therapy. The case underscores the diagnostic complexity of co-infections and the need for multimodal approaches in persistent gastrointestinal presentations.

Before a definitive diagnosis was established, the condition advanced to a liver abscess with pleuropulmonary involvement. Although the patient’s prognosis was ultimately favorable, regions lacking rapid diagnostic tools may face challenges in managing such cases, potentially leading to severe clinical outcomes.

Another unique aspect of this case is that despite early identification of a liver abscess and positive blood and pus cultures for *Salmonella*, targeted antibiotic therapy and continuous drainage failed to improve the patient's condition, which instead progressed to pulmonary and pleural involvement. After excluding drug fever and HIV-related opportunistic infections, repeated imaging provided crucial clues, and mNGS ultimately uncovered a concurrent *Entamoeba histolytica* infection, with sequences detected in both liver abscess and pleural effusion samples, confirming the amoebic origin of the invasive symptoms.

This case underscores the need for clinicians to maintain an open mindset and leverage advanced diagnostic tools like mNGS to accurately diagnose and effectively treat complex cases, thereby alleviating patient suffering.

Immunocompromised patients pose significant diagnostic challenges due to atypical presentations and the potential for co-infections. mNGS proved invaluable in identifying co-infections and guiding targeted treatment, particularly when conventional methods fail. Furthermore, comprehensive screening for immunosuppression, particularly HIV, is essential in patients with liver abscesses, even without known risk factors.

Understanding the various etiologies of liver abscesses can provide a foundation for recognizing these complex scenarios. Different liver abscess etiologies are linked to distinct risk factors, pathogenesis, and outcomes [15]. ALA often impacts immunocompetent individuals in areas with poor sanitation, caused by Entamoeba histolytica spreading from the colon to the liver via the portal vein [17]. Bacterial liver abscesses (BLA) usually result from biliary or intra-abdominal infections. Conversely, fungal liver abscesses, especially invasive liver aspergillosis (ILA), mainly occur in immunocompromised patients (e.g., post-chemotherapy or transplantation) [10]. These typically result from hematogenous spread of pulmonary infections.

With this background in mind, it is important to consider how co-infections can further complicate the clinical presentation. Previous studies have reported cases of co-infection with Entamoeba and bacteria [9], these co-infections can complicate the clinical presentation and management of liver abscesses. It is crucial for clinicians to consider the possibility of co-infections when managing patients with liver abscesses, especially in immunocompromised individuals [18]. Early and accurate diagnosis, along with appropriate treatment strategies, are essential to improve patient outcomes in such complex cases. Co-infections may be influenced by risk factors like immunocompromised states and geographical factors. Bacteria can also enhance the virulence of Entamoeba and alter the host's immune response [11], [16]. Understanding these factors can help clinicians better identify patients at risk and implement appropriate strategies.

## Conclusion

Our case highlights the need for doctors to consider co-infections in immunocompromised patients with liver abscesses unresponsive to antimicrobial therapy, emphasizing the value of advanced diagnostics like mNGS. Early detection and prompt treatment are vital for better outcomes in these complex cases.

## Abbreviations

ALA, amoebic liver abscess; mNGS, Metagenomic next generation sequencing; HU, **Hounsfield Uni**t; FLA, Fungal liver abscesses; BLA, bacterial liver abscesses; ILA, invasive liver aspergillosis; Jiaxing CDC, Jiaxing Municipal Center for Disease Control and Prevention

## Author Statement

Zhuo-Lin Zou and Zhong-Hai Shen take responsibility for the integrity of the manuscript and have contributed significantly to the work. Both authors participated in the patient's diagnosis and treatment process, analyzed the data, and were involved in drafting and revising the manuscript. Zhuo-Lin Zou was primarily responsible for the patient's clinical management and the initial draft of the manuscript, while Zhong-Hai Shen provided supervision, guided the diagnostic and treatment approach, and reviewed the manuscript thoroughly. All authors have approved the final version of the manuscript and agree with its submission to the journal. The authors confirm that this manuscript is original and has not been published elsewhere. All authors have contributed significantly to the work and agree with the submission to [IDCASE]. The authors declare no conflict of interest. The study informed consent was obtained from the patient. The data supporting the findings of this study are available from the corresponding author upon reasonable request. All authors have no other relationships or activities that could appear to have influenced the submitted work.

## CRediT authorship contribution statement

**Zhong-Hai Shen:** Writing – review & editing, Writing – original draft, Project administration, Data curation. **Zou Zhuolin:** Writing – review & editing, Writing – original draft, Funding acquisition, Data curation.

## Ethical approval

Ethical approval by the appropriate ethics review board is not required for case report submission, Written informed consent was obtained from the patient for publication of this case report and accompanying images. A copy of the written consent is available for review by the Editor-in-Chief of this journal on request.

## Funding

This work was supported by the Zhejiang Provincial Health Science and Technology Plan [Grant no.: 2025KY346].

## Consent to participate

Not applicable.

## Consent to publish

Consent to publish was not required since no personal identifiers were used in the study.

## Declaration

During the preparation of this work, the authors used ChatGPT to improve readability and language clarity. After using this tool, the authors reviewed and edited the content as needed and retained full responsibility for the content of the published article.

## Declaration of Competing Interest

The authors declare that they have no known competing financial interests or personal relationships that could have appeared to influence the work reported in this paper.

## Data Availability

Data sharing is not applicable to this article as no datasets were generated or analyzed during the current study.
